# Digital Examination vs. 4D Transperineal Ultrasound—Do They Compare in Labour Management? A Pilot Study

**DOI:** 10.3390/diagnostics14030293

**Published:** 2024-01-30

**Authors:** Friederike Exner, Rebecca Caspers, Lieven Nils Kennes, Julia Wittenborn, Tomás Kupec, Elmar Stickeler, Laila Najjari

**Affiliations:** 1 Department of Gynaecology and Obstetrics, University Hospital RWTH Aachen, 52074 Aachen, Germany; 2 Department of Economics and Business Administration, Hochschule Stralsund, 18435 Stralsund, Germany

**Keywords:** Bishop score parameters, transperineal ultrasound, labour management

## Abstract

The aim was to compare transperineal ultrasound (TPU) with parameters of the Bishop Score during the first stage of labour and evaluate how TPU can contribute towards improving labour management. Digital examination (DE) and TPU were performed on 42 women presenting at the labour ward with regular contractions. TPU measurements included the head–symphysis distance, angle of progression, diameter of the cervical wall, cervical dilation (CD) and cervical length (CL). To examine if TPU can monitor labour progress, correlations of TPU parameters were calculated. Agreement of DE and TPU was examined for CL and CD measurements and for two groups divided into latent (CD < 5 cm) and active stages of labour (CD ≥ 5 cm). TPU parameters indicated a moderate negative correlation of CD and CL (Pearson: r = −0.667; Spearman = −0.611). The other parameters showed a weak to moderate correlation. DE and TPU measurements for CD showed better agreement during the latent stage than during the active stage. The results of the present study add to the growing evidence that TPU may contribute towards an improved labour management, suggesting a combined approach of TPU and DE to monitor the latent first stage of labour and using only DE during the active stage of labour.

## 1. Introduction

The Bishop Score has been the most commonly used method for evaluating the likelihood of successful vaginal delivery after the induction of labour since 1964 [1]. A pelvic score is calculated by assessing the cervix via digital examination (DE). This score is determined by five different parameters: cervical position, consistency, effacement and dilation, as well as foetal station. It can be assumed that the higher the pelvic score, the more advanced the birth process may be [1]. Following the publication of the Bishop Score, simplifications to the Bishop Score have been made, suggesting that cervical position and consistency are not necessary for predicting successful labour induction [2,3]. Cervical dilation has been shown to be probably the most important predictor [4]. DE is not only used for assessing the cervix during the onset of labour, it is also commonly used for assessing the progress of labour [5]. Despite being well-established methods, DE and the Bishop Score have their limitations. DE is a subjective method, and the Bishop Score has a limited predictive value for successful induction of labour [6].

One examination technique which is not yet used routinely in obstetrics is transperineal ultrasound (TPU). TPU was first described by Lewin in 1976 who used it to evaluate the cervix in pregnant women [7]. Today, this method is frequently used for urologic and urogynaecologic purposes. TPU images are obtained by placing an abdominal transducer on the perineum in a sagittal plane [8].

TPU has several advantages: it is an objective and reproducible method of examination [9,10], easily accessible and less unpleasant than DE during the assessment of labour progress [11,12]. A combination of the Bishop Score and several (perineal) ultrasound parameters have been suggested in previous studies [13,14]. TPU parameters including the angle of progression (AoP), head progression distance and head–symphysis distance (HSD) were found to be associated with the duration of labour (DoL) [15] as well as the foetal head–perineum distance in combination with AoP [16] or in combination with ultrasonographically measured cervical length (CL) and foetal occiput position [17]. The labour outcome was found to be connected with the foetal head–perineum distance [18,19,20] and AoP [19]. Further, the TPU foetal head–perineum distance, cervical length, foetal head–symphysis distance, foetus entry angle and occiput position have been shown to being able to monitor the progress of labour by Khazardoost et al. [21]. Due to lower anxiety levels during TPU in the latent stage, it has also been proposed to use mainly TPU during the latent stage with as little DE as possible and to use DE during the active stage of labour [12].

The aim of the present study was to examine how TPU can contribute to labour management by examining the agreement of TPU and DE measurements, the ability of TPU and Bishop Score parameters to predict DoL and the suitability of TPU to monitor labour progress.

## 2. Materials and Methods

### Methods

Pregnant women with regular contractions presenting at the labour ward of the University Hospital Aachen, Germany, were considered for this study. Eligibility criteria included delivery at term, regular contractions and delivery in the same hospitalisation period, as well as complete ultrasound data. Women with premature rupture of the membranes, prolapse of the amniotic sac, current treatment with cervical cerclage or pessary, status post cervical conisation or who refused to participate in the study were excluded.

This study was a prospective study using consecutive sampling to recruit study participants. After giving their consent, women eligible for the study first underwent the standard procedure with the standard medical tests including cardiotocography, blood and urine sampling, foetal ultrasound and biometry, as well as blood pressure measurement. Study participants were asked to empty their bladder before ultrasound examination.

**Figure 1:**
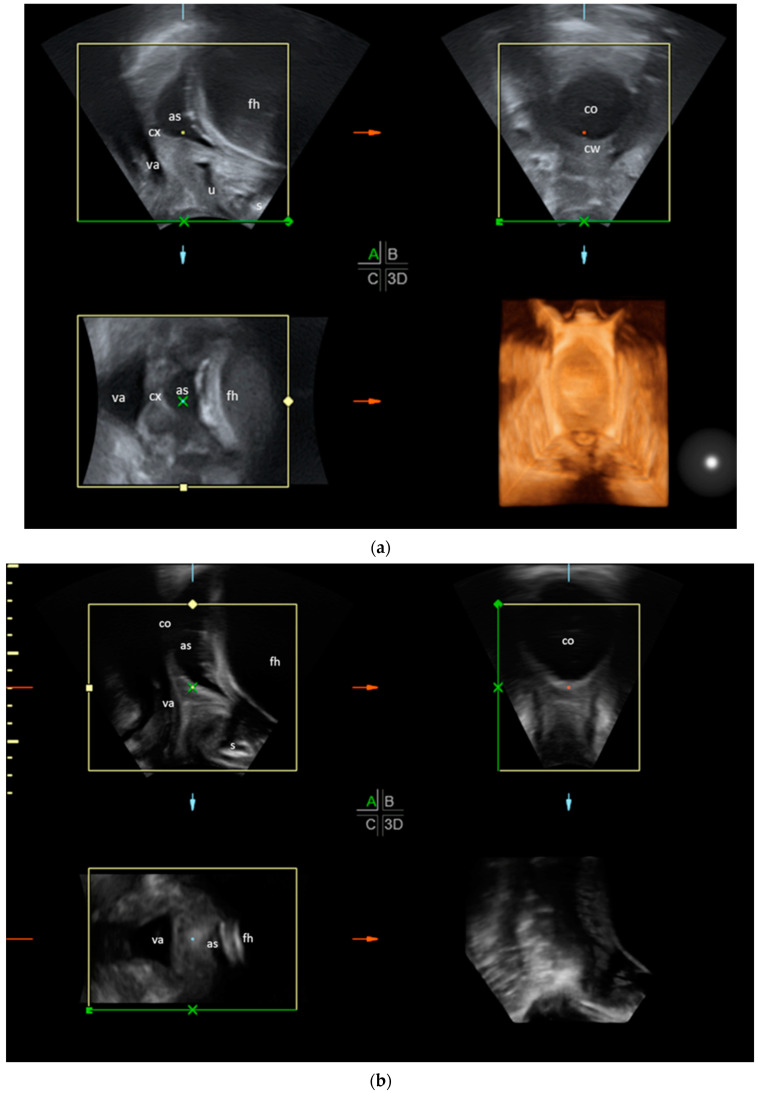
(**a**) TPU of a woman in an earlier stage of labour in sagittal plane with compilation of 4D volumes. The cervix is still closed and the cervical wall can still be clearly defined in the transverse plane. The sagittal plane is shown in the upper left, the transverse plane in the upper right, the frontal plane in the lower left and a 3D image in the lower right. as: amniotic sac; fh: foetal head; cx: cervix; va: vagina; u: urethra; s: symphysis; cw: cervical wall; co: cervical opening. (**b**) TPU of a woman in a more advanced stage of labour with a widely dilated cervix; the cervical wall is hardly visible in the transverse plane. The sagittal plane is shown in the upper left, the transverse plane in the upper right, the frontal plane in the lower left and a 3D image in the lower right. as: amniotic sac; fh: foetal head; cx: cervix; va: vagina; u: urethra; s: symphysis; co: cervical opening.

TPU measurements were performed by one experienced examiner with a DEGUM II certificate. Measurements were conducted with an ultrasound scanner (GE Voluson 730 Expert) using a Curves-linear-Array-probe (3.5–5 MHz). Four dimensional ultrasound volumes were acquired by positioning the probe on the perineum in the sagittal plane after separating the labia, as described by Dietz et al. [8]. The transverse and frontal plane as well as a 3D image were generated by the ultrasound unit using the volumes gained in the sagittal plane (Figure 1a,b).

**Figure 2:**
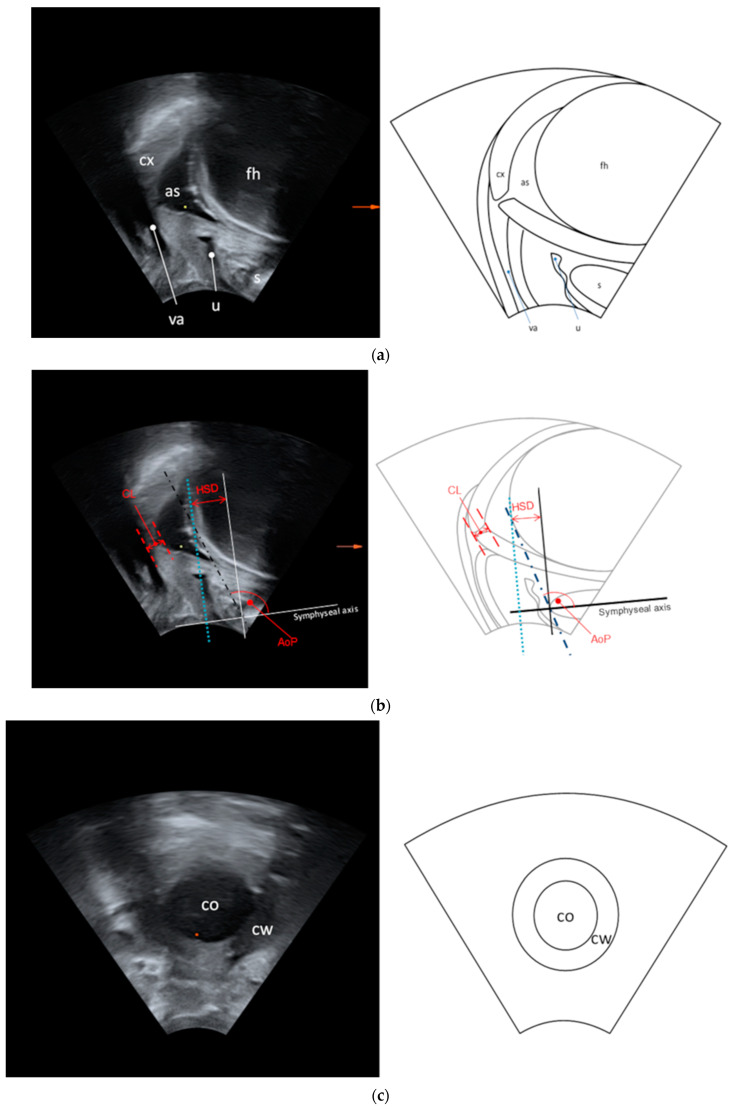

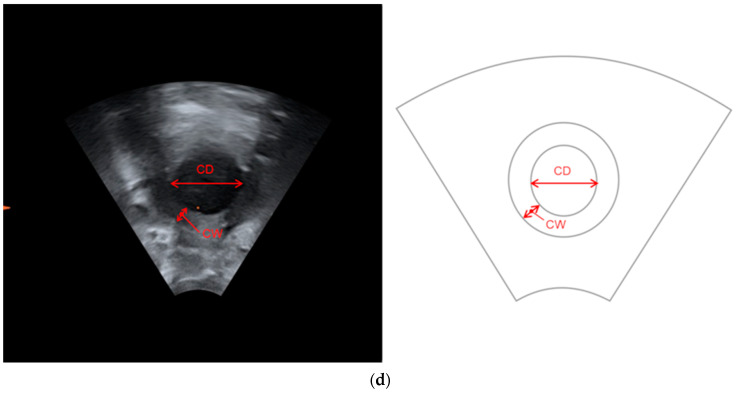
(**a**) Ultrasound image and drawing in the sagittal plane. as: amniotic sac; fh: foetal head; cx: cervix; va: vagina; u: urethra; s: symphysis. (**b**) Ultrasound measurements in the sagittal plane. AoP: angle of progression; HSD: head–symphysis distance; CL: cervical length. (**c**) Ultrasound image and drawing in the transverse plane.co: cervical opening; cw: cervical wall. (**d**) Ultrasound measurements in the transverse plane. CD: cervical dilation; CW: diameter of the cervical wall.

TPU measurements were performed in the sagittal and transverse plane with the software 4D View (Version 6.0). In the sagittal plane, the symphysis, foetal head, amniotic sac, cervix, vagina and urethra were displayed in one ultrasound image (Figure 2a), enabling measurements of the AoP, HSD and CL.

For AoP and HSD, a line drawn through the long symphyseal axis served as a reference point. AoP was defined as the angle between the symphyseal axis and a tangent from the most inferior point of the symphysis to the contour of the foetal scull, as described by Barbera et al. [22]. HSD was defined as the distance between a reference line vertical to the long symphyseal axis placed through the symphyseal margin and another line vertical to the long symphyseal axis, tangential to the foetal scull, as described by Dietz et al. [23] (Figure 2b). The transverse plane displayed the cervical opening and the cervical wall, thus permitting measurements of the diameter of the cervical wall (CW) and cervical dilation (CD) (Figure 2c,d).

DE was carried out immediately after the ultrasound examination by an experienced midwife on duty (eight different midwives in total). The midwife was blinded to the TPU results. For the assessment of the cervix, DE was performed with the woman in the lithotomy position. The following parameters of the Bishop Score were examined via DE: cervical dilation, cervical effacement, foetal station, cervical position and the consistency of the cervix. In this study, we chose to exclude the consistency of the cervix as it cannot be easily measured by TPU and has been shown to probably be the least useful element of the Bishop Score [4]. The Bishop Score without cervical consistency is hereafter called the modified Bishop Score (mBS), which was used as a reference (displayed in Table 1).

The results were analysed with descriptive statistics. The following outcomes were assessed:

To find out if TPU measurements can monitor the progress of labour, we calculated the correlation of the TPU parameters using Pearson’s and Spearman’s correlation coefficients. A moderate correlation was defined as r = 0.4–0.69 [24]. The results will give insight into how single TPU parameters are related to each other with the assumption that if there is an expected association between different parameters, they are more likely suitable to monitor labour progress.

To compare DE and TPU measurements, Bland–Altman plots and Lin’s concordance correlation coefficients were calculated for both CL and CD. A moderate correlation was defined as ρ_c_ = 0.41–0.60 [25]. The results will give insights into the agreement of both measuring techniques.

For comparison of DE and TPU measurements during the first active and latent stage of labour, we categorised the TPU measurements into two groups, differentiating the latent stage from the active first stage of labour according to the definition of the WHO (latent stage: CD < 5 cm and active stage: ≥ 5 cm) [26]. The agreement of DE and TPU measurements in both groups were again evaluated using Bland–Altman plots and Lin’s concordance correlation coefficients. The results will give an indication of whether there is a difference between usability of TPU during the latent and active stage.

The ability of TPU and mBS to predict DoL (<6 h yes/no and <12 h yes/no) was investigated by logistic regression and their resulting receiver operating characteristic (ROC) curves. For better description purposes, the area under the receiver operating characteristic curve (AUC) was defined as fair at a threshold of 0.7 while acknowledging that AUC values should best not be labelled [27]. The ability of combined TPU parameters to predict DoL was calculated with a multivariate logistic regression model including all parameters of TPU. They were then compared to results of a logistic regression of the total mBS on DoL.

All statistical analyses were performed in an explorative manner using the statistical software R (version R 4.0.2) [28].

## 3. Results

**Figure 3:**
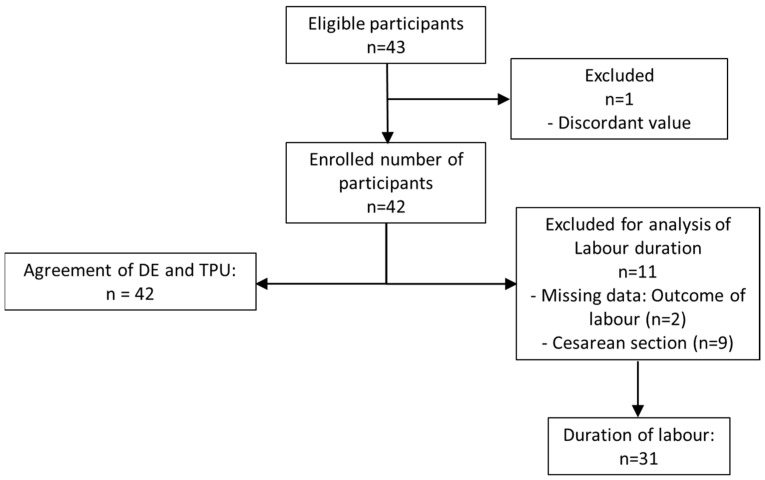
Flow of participants.

Of the 43 eligible women, 1 was excluded due to discordant values (time until birth was 52 h, despite initial CD of 6 cm). The remaining 42 participants were enrolled in the study. The data of 42 and 31 participants were analysed for agreement of DE and TPU measurements. Further, the association of DoL with TPU and DE, respectively, was examined (Figure 3).

Baseline demographics and clinical characteristics of the study population are displayed in Table 2.

### 3.1. Correlation of TPU Parameters

As shown in Table 3, CD and CL showed the strongest (negative) correlation. All other parameters showed weak to moderate correlations.

### 3.2. Comparison of DE Measurements with TPU

When comparing DE and TPU, TPU measurements appeared to result in longer CL compared to DE measurements, which indicated poor agreement (mean difference DE—TPU: −1.09 cm; limits of agreement 0.745 cm and −2.61 cm, respectively; Lin’s concordance coefficients (ρ_c_) = 0.25; 95% confidence interval (CI): 0.12–0.37). Short cervical lengths seemed to be measured longer with DE while larger lengths seemed to be measured longer with TPU.

CD was measured to be around 0.12 cm wider by DE than by TPU, showing moderate agreement of both methods (limits of agreement: 2.95 cm and −2.42 cm, respectively; ρ_c_ = 0.48; 95% CI: 0.21–0.68).

### 3.3. Comparison of DE Measurements with TPU during Active vs. Latent Stage of Labour

The measurements for DE vs. TPU for latent vs. active first stage of labour are shown in Table 4.

**Figure 4:**
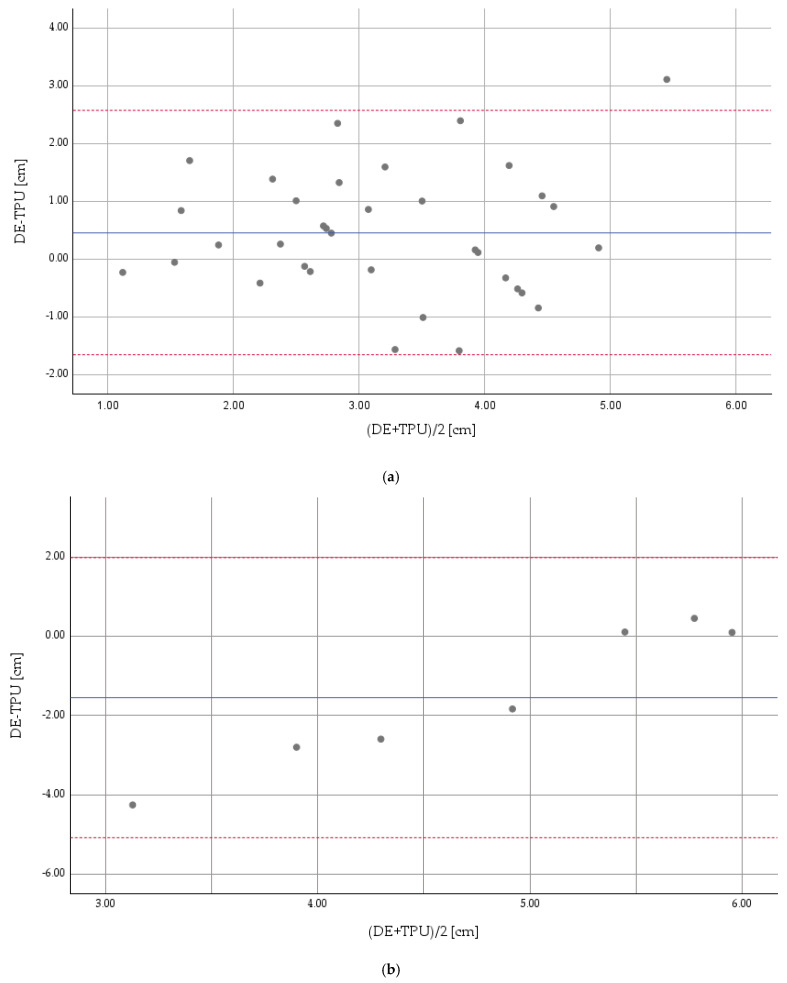
(**a**): Concordance of DE and TPU measurements for the latent stage (CD < 5 cm) *. * Reference measuring method: TPU. Continuous line: mean difference. Dotted lines: limits of agreement. The Bland–Altman diagram shows an average difference between DE and TPU examination of 0.45 cm, with limits of agreement of 2.57 cm and −1.66 cm, respectively. CD: cervical dilation; DE: digital examination; TPU: transperineal ultrasound. (**b**) Concordance of DE and TPU measurements for the active first stage (CD ≥ 5 cm) *. * Reference measuring method: TPU. Continuous line: mean difference. Dotted lines: limits of agreement. The Bland–Altmann diagram shows an average difference between DS and TPU examination of −1.55 cm, with limits of agreement of 1.98 cm and −5.08 cm, respectively. CD: cervical dilation; DE: digital examination; TPU: transperineal ultrasound.

When comparing DE and TPU measurements, we found CD measurements moderately correlated during the latent stage of labour (TPU CD < 5 cm, Lin’s concordance coefficient ρ_c_ = 0.52; 95%—CI: 0.28–0.73), Figure 4a.

For DE vs. TPU measurements performed during the active first stage of labour (TPU CD ≥ 5 cm), CD showed no agreement (Lin’s concordance coefficient ρ_c_ = 0.085; 95%—CI: −0.048–0.22), Figure 4b.

### 3.4. Prediction of DoL

As shown in Table 5, both TPU parameters combined and the mBS in total showed fair accuracy in predicting delivery within the next 12 h, with TPU showing better numerical values. Both methods indicated a weaker accuracy for predicting delivery within the next 6 h. Concerning the individual parameters of TPU and mBS, only digitally examined CD showed fair accuracy in predicting delivery within the next 12 h, similar to that of mBS in total and TPU combined.

## 4. Discussion

Our study confirmed that in the early stages of labour, TPU parameters may be suitable to monitor the progress of labour and that CD measurements performed by DE and TPU are in moderate agreement. We found that the ability to predict DoL within 12 h is similar for DE and TPU.

With regard to the correlation of TPU parameters, due to acceptable (moderate) correlations of most TPU parameters, we conclude that it is possible to monitor labour progress with TPU. Other studies support this observation. Chan et al. reported good correlation between AoP and head–perineum distance [16]. Ciaciura-Jarno et al. confirmed the ability of TPU to track labour progress in their study and found good correlations between HSD and AoP [29]. Hassan et al. also found that TPU was appropriate to monitor the progress of labour by assessing head descent and cervical dilation [30].

Our results for the agreement of DE and TPU parameters show that for CL, there was poor agreement between the DE and TPU measurements. This could be due to our small sample size, as other studies have found better agreement [31]. However, it cannot be concluded that TPU measurements were more imprecise, as the actual length of the cervix is unknown. CL measured by TPU has been shown to be reproducible [10] and to be in good agreement with transvaginal measurements of CL [32]. Therefore, we conclude that despite poor agreement in our study, CL measured by TPU could still be valuable in labour management.

When looking at the agreement of CD measured by DE and TPU, we found better agreement in the latent stage group (CD < 5 cm). This is also supported by other studies. Usman et al. found TPU measurements to be unreliable for CD larger than 4 cm [33]. Benediktsdottir et al. reported ultrasound to be unable to measure CD > 8 cm [34]. Kwan et al. reported overall good agreement of DE and TPU measurements for CD while also stating that the ability of TPU to measure CD decreased with growing CD [35]. This suggests that TPU is more suitable for the latent stage than for the later stages of labour.

Generally, most studies found better correlations of CD measured by DE and TPU. We only found moderate correlations and suspect that this is due to the small sample size of our study. One could argue that the mean difference of 0.12 cm between both measuring techniques for CD in the latent stage group still appears to be acceptable. A meta-analysis showed that correlation of TPU and DE was higher in multigravida women than in nulliparous women [36]. As we did not differentiate between nulliparous and multiparous women, this may also have led to lower correlations.

As in the present study, other studies found CD to be frequently measured wider by DE than by TPU [30]. This can be explained by the increasing flexibility of the cervix during labour progress, which leads to additional cervical dilation during DE, leading to wider measurements.

As for the prediction of DoL, CD as a single parameter and mBS parameters combined seemed to have a similar ability to predict delivery within 12 h. This implicates an important role of CD within the mBS parameters. The observations of previous studies also support this conclusion as they have shown CD to be the most important predictor of the Bishop Score for successful labour induction (defining successful induction as vaginal delivery within 12 and 24 h of induction) [4].

Contrary to our study, other studies found a better ability of single TPU parameters to predict DoL. Ghi et al. examined AoP, progression distance (PD) and HSD with TPU. They found that greater values of AoP and PD at the beginning of the active stage correlated with a shorter time to delivery. Also, they found a direct relationship between HSD and the duration of the active second stage [15]. Chan et al. reported that the AoP and head–perineum distance could predict the time to delivery during the active stage of labour [16]. Hjartardóttir et al. found CD measured by TPU during the active stage to be significantly associated with DoL [37]. These differences may result from the fact that most of our examinations were performed during the latent stage, whereas most other studies performed their examinations during the active stage of labour.

However, our study did show a similar ability of mBS and TPU parameters combined to predict delivery within 6 h and 12 h, with better numerical values in both cases for TPU. Due to the small sample size of the present study, a statement concerning the specific cut off values for each parameter indicating delivery within 6 or 12 h cannot be made.

One of the main limitations of this study is the small sample size. Also, no differentiation between nulli- and multiparae was made. Further, eight different midwives performed DE, in contrast to only one ultrasound examiner. Although the examinations were carried out right after each other, time intervals between both examinations were not recorded. The mBS we used has not been validated in earlier studies.

Due to the small sample size, this study should be interpreted as a pilot study, being a possible inspiration for further studies.

So far, the most common method of monitoring labour progress has been via DE. However, there is increasing evidence that other examination methods to monitor labour progress may be useful and helpful. TPU has shown to be reproducible [10], significantly less painful than DE and to lessen anxiety levels during the latent stage of labour [12]. Higher anxiety levels have been shown to be associated with a longer time to delivery [38] and higher degrees of pain [39]. These findings suggest that reducing anxiety levels could have a positive influence on the labour process and labour outcomes, making TPU a suitable examination method. Also, TPU is a widely accessible and objective method. It is able to provide a lot of information in just one picture, including the filling of the bladder, amniotic sac and foetal position. A breech position or umbilical cord prolapse can easily be detected. Further, routine use of TPU could reduce the number of DEs needed to monitor the progress of labour during the latent stage. It is also a method that can be performed by midwives after appropriate training [40]. A disadvantage of TPU is its inability to dilate the cervix, thus making it unsuitable for use in later stages of labour. The results of the present study add to the growing evidence that TPU may contribute towards improved labour management during the early stages of labour. Therefore we support the suggestion made in previous studies to reduce DE during the latent stage of labour by additionally using TPU to monitor labour progress. During the active stage, DE should still routinely be used.

## 5. Conclusions

In this study, we examined the agreement of TPU and DE measurements, the ability of TPU and mBS parameters to predict DoL and the suitability of TPU to monitor labour progress. Our study showed that monitoring labour progress with TPU is possible, being more suitable during the latent stage of labour than during later stages. This suggests that routine use of TPU in addition to DE during the latent stage could contribute to labour management by reducing the number of DEs and anxiety levels and adding an objective component. Further, combined TPU parameters and mBS show a similar ability to predict labour within 12 h. Due to the small sample size of this study, potential cutoff values for a better prediction of DoL could not be made. This, however, could be the object of larger studies in future. Also, the validity and practicability of this approach should be tested in future studies.

## Figures and Tables

**Table 1:** Modified Bishop Score. Total points: 11.

Points	0	1	2	3
Cervical dilation [cm]	0	1–2	3–4	5–6
Cervical effacement [%]	0–30	40–50	60–70	≥80
Foetal station	−3	−2	−1/0	+1/+2
Cervical position	Posterior	Middle	Anterior	

**Table 2:** Baseline demographics and characteristics of the study population.

Maternal Characteristics:	Median (Range)	Labour Characteristics:	*n*
Age (years)	33 (20–42)	Spontaneous vaginal delivery	28 (67%)
Parity	1 (0–5)	Operative vaginal delivery	5 (12%)
Gestation week	39 + 5 (37 + 3 − 41 + 3)	Cesarean section	9 (21%)

**Table 3:** Correlation of individual TPU parameters. AoP: angle of progression; CL: cervical length; CD: cervical dilation; CW: diameter of the cervical wall; HSD: head–symphysis distance; TPU: transperineal ultrasound.

TPU Parameters	AoP	CL	CD	CW
HSD	0.598 ^†^	0.493 ^†^	−0.286 ^†^	0.124 ^†^
	0.573 ^††^	0.440 ^††^	−0.261 ^††^	0.218 ^††^
AoP		0.508 ^†^	−0.172 ^†^	0.170 ^†^
		0.351 ^††^	−0.127 ^††^	0.200 ^††^
CL			−0.667 ^†^	0.450 ^†^
			−0.611 ^††^	0.570 ^††^
CD				−0.530 ^†^

^†^ Pearson’s correlation coefficient; ^††^ Spearman’s correlation coefficient.

**Table 4:** CD measurements performed by DE and TPU. Groups were divided into latent and active stage of labour using CD measurements by TPU (TPU CD < 5 cm and ≥5 cm). CD: cervical dilation; DE: digital examination; SD: standard deviation; TPU: transperineal ultrasound.

Stage of Labour	Examination Method	Mean (SD) [cm]	Median (Min; Max) [cm]
Latent stage CD < 5 cm *n* = 35	DE	3.43 (1.207)	3.00 (1.00; 7.00)
	TPU	2.97 (1.155)	2.65 (0.80; 4.85)
Active first stage CD ≥ 5 cm *n* = 7	DE	4.00 (1.936)	4.00 (1.00; 6.00)
	TPU	5.55 (0.253)	5.55 (5.26; 5.90)

**Table 5:** Ability of mBS and TPU parameters to predict DoL. AUC: area under the curve; DoL: duration of labour; mBS: modified Bishop Score; TPU: transperineal ultrasound.

		Predicted DoL < 6 h	Predicted DoL < 12 h
Delivery within the predicted timeframe	Yes	*n* = 18	*n* = 21
	No	*n* = 13	*n* = 10
Method	Parameter	AUC	AUC
DE	Cervical effacement ^†^	0.542	0.598
	Cervical postion ^†^	0.450	0.530
	Cervical dilation ^†^	0.616	0.712
	Foetal station ^†^	0.555	0.540
	mBS total ^†^	0.599	0.717
TPU	Head–Symphysis distance ^†^	0.542	0.490
	Angle of progression ^†^	0.513	0.389
	Cervical length ^†^	0.498	0.480
	Cervical dilation ^†^	0.513	0.485
	Cervical wall ^†^	0.544	0.649
	Combined parameters ^††^	0.622	0.722

^†^ univariate approach; ^††^ multivariate approach (logistic regression model).

## Data Availability

The data presented in this study are available on request from the corresponding author.
